# Cardiovascular magnetic resonance-derived metrics as diagnostic markers for left ventricular outflow tract obstruction in hypertrophic cardiomyopathy

**DOI:** 10.1016/j.jocmr.2026.102691

**Published:** 2026-01-14

**Authors:** Georgios M. Alexandridis, Stephan A.C. Schoonvelde, Anne J. Koppelaar, Peter-Paul Zwetsloot, Ricardo P.J. Budde, Isabella Kardys, Arend F.L. Schinkel, Rudolf A. de Boer, Michelle Michels, Alexander Hirsch

**Affiliations:** a Department of Cardiology, Cardiovascular Institute, Thorax Center, Erasmus MC, Rotterdam, the Netherlands; b Erasmus MC Graduate School, Erasmus MC, Rotterdam, the Netherlands; c Department of Radiology and Nuclear Medicine, Erasmus MC, Rotterdam, the Netherlands; d Netherlands Heart Institute, Utrecht, the Netherlands

**Keywords:** Hypertrophic cardiomyopathy, Left ventricular outflow tract obstruction, Cardiovascular magnetic resonance, Doppler echocardiography

## Abstract

**Background:**

This study aims to assess the utility of cardiovascular magnetic resonance (CMR)-derived measurements as diagnostic indicators for left ventricular outflow tract obstruction (LVOTO) in hypertrophic cardiomyopathy (HCM) patients, using Doppler echocardiography as the reference method.

**Methods:**

In this single-center retrospective cross-sectional study, adult HCM patients with transthoracic echocardiography and CMR within a 6-month time window were enrolled. Doppler LVOT gradient was measured both at rest and under provocative maneuvers. LVOTO was defined as a gradient ≥30 mmHg. The total cohort was randomly divided into a training (80%) and validation (20%) cohorts, maintaining the same proportions of patients with and without LVOTO in both cohorts. In CMR, all measurements were performed in the three-chamber balanced steady-state free precession cine images. CMR metrics were examined in relation to LVOTO by means of multivariable logistic regression models.

**Results:**

Four hundred forty-eight adult HCM patients (67% (301/448) males, median (25th-75th percentile) age 55 (45-62) years) were enrolled. LVOTO was present in 42% (186/448) HCM patients. For identifying LVOTO, a model including the minimum distance between the mitral leaflet tip and the interventricular septum indexed to body surface area (minimum MV-IVSi distance), left ventricular (LV) stroke volume, and signal intensity ratio LVOT/LV showed an outstanding discriminatory ability in the validation cohort with an area under the curve (AUC) of 0.91 (95% confidence interval (CI) 0.85-0.97). The univariable model of the minimum MV-IVSi distance showed an AUC of 0.88 (95%CI 0.81-0.95). An MV-IVSi distance ≤6.5 mm/m^2^ yielded a specificity of 94% and a positive predictive value of 89%, and >9.0 mm/m^2^, a sensitivity of 97% and a negative predictive value of 97%. The minimum MV-IVSi distance showed excellent intra- and inter-observer reproducibility with an intraclass correlation coefficient of ≥0.95.

**Conclusion:**

CMR-derived parameters, particularly the minimum MV-IVSi distance, can accurately identify LVOTO in HCM patients and easily be integrated into a standard CMR analysis.

## Graphical abstract

**Figure:**
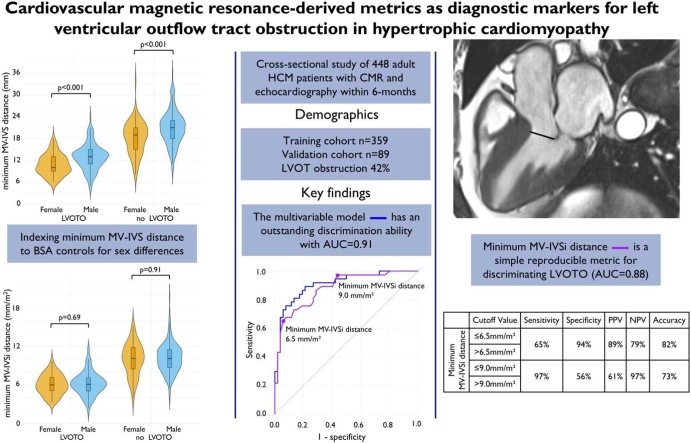
*AUC* area under the curve, *BSA* body surface area, *HCM* hypertrophic cardiomyopathy, *LVOTO* left ventricle outflow tract obstruction, *Minimum MV-IVSi distance* minimum mitral valve leaflet tip to interventricular septum distance indexed.

## 1 Introduction

Hypertrophic cardiomyopathy (HCM) is a relatively common inherited heart muscle disease, with a prevalence ranging from 1:200 to 1:500 in a general population of young adults [1], [2], [3]. An important pathophysiological component of HCM is the left ventricular outflow tract (LVOT) obstruction (LVOTO), defined as peak instantaneous Doppler LVOT gradient ≥30 mmHg, either at rest or under provocative maneuvers [4], [5], [6]. LVOTO is associated with symptoms and an increased risk of heart failure and sudden cardiac death, affecting up to 70% of HCM patients [6], [7], [8], [9], [10].

Three main mechanisms are considered to be responsible for the LVOTO: (i) left ventricular (LV) hypertrophy, including the basal septum, which narrows the LVOT, causing abnormal blood flow; (ii) mitral valve abnormalities, including longer leaflets and systolic anterior motion (SAM) of the leaflets, and (iii) LV chamber morphology, such as anterior displacement of the papillary muscles and accessory chordae [9], [11], [12], [13].

The introduction of novel disease-specific treatments, such as cardiac myosin inhibitors, has shown promising results in improving exercise capacity and quality of life in patients with LVOTO, underlining the importance of early and accurate diagnosis [10], [14], [15], [16]. Doppler echocardiography is currently the gold standard method for diagnosing LVOTO [4], [5], [8]. The LVOT gradient should also be measured under provocative maneuvers, in line with current guideline recommendations, to reveal potential latent obstruction [4], [5], [8]. While provocative maneuvers such as Valsalva or exercise testing are clinically valuable, their use in daily practice may vary, as some HCM patients face challenges in adhering to maneuver instructions, contributing to the underdiagnosis of LVOTO [9].

Cardiovascular magnetic resonance (CMR) is performed more and more in patients with HCM and is recommended at their baseline assessment [4]. However, CMR does not reliably capture direct outflow hemodynamic measurements [4], [8], [17]. Although previous studies have aimed to detect LVOTO indirectly by capturing the underlying pathophysiological and spatial alterations [12], [17], [18], [19], [20], [21], [22], there is still a need for easy-to-measure and reliable diagnostic metrics that can be used in daily clinical practice. Therefore, this study aims to assess the utility of CMR-derived metrics as diagnostic indicators for LVOTO in HCM patients.

## 2 Methods

### 2.1 Study population

For this single-center cross-sectional study, patients included in the HCM registry at the Erasmus MC were screened. All individuals gave written informed consent for inclusion in this local registry, for which local Institutional Review Board approval was obtained. HCM was diagnosed according to the European Society of Cardiology guidelines and was defined by a maximal wall thickness (MWT) ≥15 mm in any myocardial segment that could not be explained solely by loading condition, or ≥13 mm in first-degree relatives of HCM patients [4]. Adult HCM patients of all phenotypes, including isolated apical HCM, were eligible for the study if they had undergone both transthoracic echocardiography (TTE) and CMR, with a time interval of <6 months between the two imaging modalities. The TTE had to be performed at our center, and the LVOT pressure gradient was measured at rest and under provocative maneuvers. In cases where multiple TTEs or CMRs were available, the pair with the shortest time difference was selected. TTEs with missing data or inadequate imaging quality were excluded. Patients with a mechanical mitral valve or who had undergone septal reduction therapy within 3 months before or between the two imaging modalities were excluded from the study.

### 2.2 Echocardiography

All patients underwent TTE by dedicated sonographers at our center, where the peak instantaneous Doppler LVOT gradient was measured at rest and under provocative maneuvers. LVOTO was defined as a peak instantaneous Doppler gradient of ≥30 mmHg. Provocable LVOTO was defined as a gradient of <30 mmHg at rest that increased ≥30 mmHg under provocative maneuver.

### 2.3 Cardiovascular magnetic resonance

**Fig. 1:**
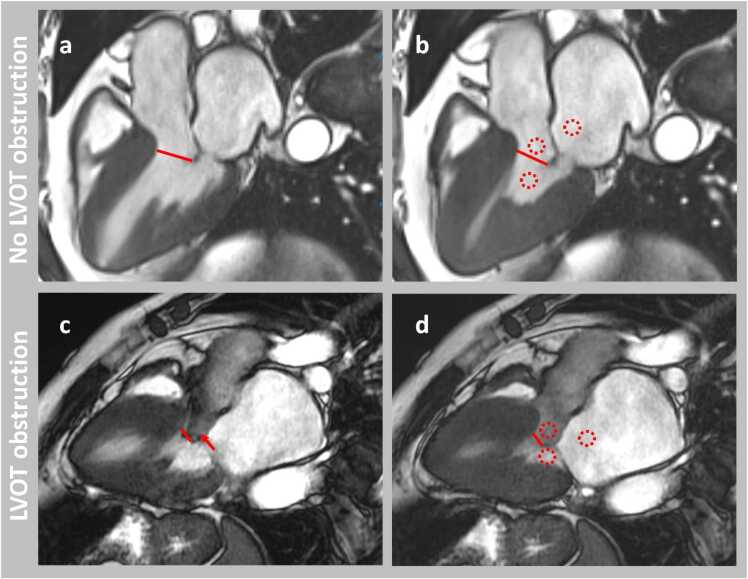
Measurements in the three-chamber balanced steady-state free precession cine images in hypertrophic cardiomyopathy patients. Top row: three-chamber balanced steady-state free precession (bSSFP) cine images of a hypertrophic cardiomyopathy (HCM) patient with no left ventricular outflow tract (LVOT) obstruction in mid-systole (a) and end-systole (b). Bottom row: three-chamber bSSFP cine images of an HCM patient with LVOT obstruction in mid-systole (c) and end-systole (d). Red arrow: presence of flow artifact in LVOT during systole; red circles: regions of interest (ROI) for signal intensity measurements. Mean signal intensity was measured manually in ROIs of approximately 0.5 cm^2^ at end-systole in the LVOT, left ventricle, and left atrium (1–2 cm above the mitral valve). In cases of mitral regurgitation, the ROI in left atrium was adjusted to avoid the mitral regurgitation jet; red line: the shortest distance between the mitral valve leaflet tip and the interventricular septum. Left ventricular volumes were measured on the short-axis cine images using standard methods, including the papillary muscles and trabeculations in the blood volume. LV stroke volume was calculated by subtracting LV end-systolic volume from LV end-diastolic volume. *LV* left ventricular

The CMR images were analyzed by a single observer (G.M.A.) blinded to the TTE results, using the Medis Suite Cardiac Software version 4.0 (Medis Medical Imaging, Leiden, The Netherlands). All measurements were performed in the three-chamber balanced steady-state free precession (bSSFP) cine images. The following parameters were measured during diastole: the MWT of the intraventricular septum (IVS), the basal septal thickness, the aortic valve diameter, the anterior and posterior mitral valve leaflet length, and the LV-aortic root angle. The LV-aortic root angle was defined as the angle between a line connecting the LV apex to the midpoint of the mitral valve and a line passing through the center of the aortic root. In mid- and end-systole, the distance between the mitral leaflet tip and the IVS (MV-IVS) was measured. The minimum MV-IVS distance was defined as the smallest value of these two. Additionally, the mean signal intensity (SI) in the LVOT, LV, and left atrium (LA) was measured in end-systole, and the SI ratios of the LVOT/LV and LVOT/LA were calculated. These measurements were based on a previous publication in patients with aortic stenosis where the SI ratio on bSSFP was a good marker for stenosis severity [23]. Moreover, flow artifacts in the LVOT and SAM of mitral valve leaflets were visually assessed during systole as present or absent. Detailed definitions can be found in Supplementary Table S1, and examples are shown in Fig. 1. Finally, LV volumes and ejection fraction were measured on the short-axis cine images using standard methods, including the papillary muscles and trabeculations in the blood volume. LV volumes and MV-IVS distances were corrected for body surface area, determined by the Du Bois formula.

To assess intra- and inter-reader reproducibility, a random sample of 30 CMR studies was reanalyzed 3 months later by the same observer (G.M.A.) and by a second observer (A.J.K.) blinded to the results.

## 3 Statistical analysis

Continuous variables were described with median and 25th–75th percentiles and percentages were used for categorical variables. Group comparisons were performed using the Mann-Whitney U test for continuous variables and the chi-square test or Fisher’s exact test for categorical variables. The total cohort was randomly divided into a training (80%) and validation (20%) cohorts, maintaining the same proportions of patients with and without LVOTO (defined as a gradient of ≥30 mmHg at rest or during provocation) in each cohort. Associations between the CMR parameters and LVOTO were assessed in the training cohort using logistic regression. First, univariable analyses were performed. Then, for multivariable analysis, backward elimination was performed, starting with a full model of 17 parameters. At each step, the variable with the highest p-value was eliminated, until p < 0.05 was reached for all remaining variables. Multicollinearity was evaluated by examining the variance inflation factors, and all of them had a value <5. The performance of the models built in the training cohort was evaluated in both the training and validation cohorts, using receiver operating characteristic (ROC) curve analysis, with the area under the curve (AUC) and corresponding 95% confidence interval (CI) as a measure of discrimination. The discriminatory ability of the models was also examined in a subset of both cohorts, after excluding patients with LVOTO at rest, to evaluate the ability of the models to identify provocable LVOTO specifically. Evaluation of multiple cut-off values for minimum MV-IVSi (indexed) and minimum MV-IVS (non-indexed) distances for diagnosing LVOTO was performed to identify thresholds optimized for sensitivity and specificity. Intra- and inter-reader reproducibility was visualized using Bland-Altman plots and reported using intraclass correlation coefficients (ICC) with a two-way random effects model for absolute agreement for continuous variables and Cohen’s kappa for categorical variables.

For all analyses, two-tailed p-values <0.05 were considered statistically significant. Analyses were conducted using R (version 4.5.0; R Core Team, 2025) within RStudio (version 2023.12.0+369; RStudio: Integrated Development Environment for R.Posit Software, PBC, Boston, Massachusetts)

## 4 Results

**Fig. 2:**
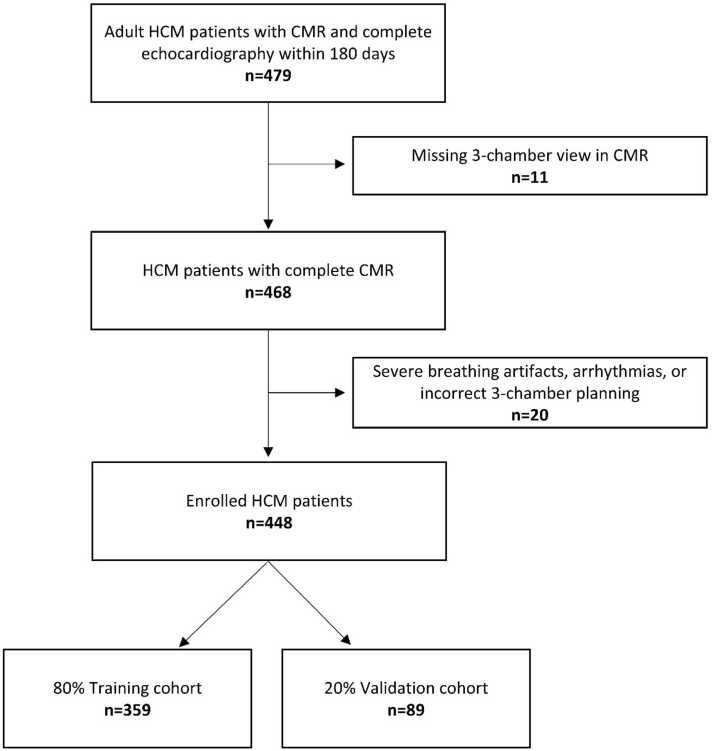
Study flowchart. *CMR* cardiovascular magnetic resonance, *HCM* hypertrophic cardiomyopathy

A total of 479 HCM patients with a CMR and TTE from 2003 to 2024 were eligible for inclusion. In 11 patients, the three-chamber cine was missing. Of the 468 remaining patients, 4.3% (20/468) were excluded due to inadequate imaging quality (severe breathing artifacts/arrhythmias) or incorrect three-chamber planning. Finally, 448 HCM patients were included. The cohort was randomly divided into a training cohort (n = 359) and a validation cohort (n = 89), maintaining the same proportions of patients with and without LVOTO in both cohorts (Fig. 2).

### 4.1 Demographic and imaging characteristics

The demographic characteristics of the included patients are summarized in Table 1 and Supplementary Table S2. The median (25th–75th percentile) age was 55 (45–62) years, and 67% (301/448) were males. Genotype data were available for 90% (402/448) of the patients, with a (likely) pathogenic gene variant identified in 53% (214/402) of them. The median time between TTE and CMR was 60 (35–92) days. LVOTO was present in 186 patients (42% (186/448)), of whom 149 were in the training cohort and 37 in the validation cohort. Of note, 65 (35% (65/186)) had only provocable LVOTO, including 53 in the training and 12 in the validation cohorts.

**Table 1:** Clinical, genetic, and imaging characteristics of patients in the training and validation cohorts.

	Total (n = 448)	Training cohort (n = 359)	Validation cohort (n = 89)	p-value
Age (y)	55 (45–62)	54 (44–62)	57 (48–63)	0.14
Male sex	301 (67)	249 (69)	52 (58)	0.07
Weight (kg)	84 (73–94)	84 (74–94)	83 (69–90)	0.08
Height (cm)	176 (168–183)	176 (169–183)	173 (165–181)	0.04
Body surface area (m^2^)	2.0 (1.9–2.1)	2.0 (1.9–2.1)	2.0 (1.8–2.1)	0.04
History of septal reduction therapy (>3 months before imaging)	18 (4)	16 (4)	2 (2)	0.55
Time between CMR and echocardiography (days)	60 (35–92)	60 (36–92)	59 (28–92)	0.45
*Genotype testing*	(n = 402)	(n = 326)	(n = 76)	0.19
(Likely) pathogenic gene variant	214 (53)	175 (54)	39 (57)	0.81
Gene				0.90
MYBPC3	151 (71)	124 (71)	27 (69)	–
MYH7	28 (13)	23 (13)	5 (13)	–
MYL2	12 (6)	9 (5)	3 (8)	–
Other	23 (11)	19 (11)	4 (10)	–
*Transthoracic echocardiography*				
LVOT gradient at rest (mmHg)	9 (5–39)	8 (5–37)	10 (6–45)	0.61
LVOT gradient at provocation (mmHg)	14 (7–78)	13 (7–81)	17 (7–67)	0.69
LVOT obstruction	186 (42)	149 (42)	37 (42)	-
*Cardiovascular magnetic resonance*				
LVEDVi (mL/m^2^)	81 (72–94)	82 (72–94)	80 (74–91)	0.62
LVESVi (mL/m^2^)	31 (25–39)	32 (25–39)	30 (26–36)	0.39
LVSVi (mL/m^2^)	50 (43–56)	50 (44–57)	50 (43–56)	0.83
LVEF (%)	62 (56–67)	61 (56–66)	62 (55–67)	0.45
LVEF <50%	40 (9)	33 (9)	7 (8)	0.85
Maximal wall thickness IVS (mm)	18 (16–21)	18 (16–22)	17 (15–20)	0.09
Basal septal thickness (mm)	11 (10–13)	12 (10–14)	11 (10–13)	0.01
Aortic valve diameter (mm)	24 (21–26)	24 (21–26)	24 (21–26)	0.68
Presence of flow artifact during systole in LVOT	219 (49)	173 (48)	46 (52)	0.64
Systolic anterior motion of the mitral valve	173 (39)	139 (39)	34 (38)	1.00
AMVL length (mm)	27 (24–29)	27 (25–29.5)	26 (23–29)	0.09
PMVL length (mm)	15 (14–18)	15 (14–18)	15 (13–18)	0.87
MV-IVS mid-systole (mm)	18 (13–23)	18 (14–23)	18 (13–22)	0.18
MV-IVSi mid-systole (mm/m^2^)	9.2 (6.7–11.7)	9.3 (6.8–11.7)	9 (6.6–11.2)	0.35
MV-IVS end-systole (mm)	17 (13–21)	17 (13–22)	16 (13–20)	0.16
MV-IVSi end-systole (mm/m^2^)	8.6 (6.7–10.8)	8.8 (6.7–10.8)	8.2 (6.8–10.1)	0.29
Minimum MV-IVS distance (mm)	16 (13–21)	17 (13–21)	15 (12–20)	0.12
Minimum MV-IVSi distance (mm/m^2^)	8.3 (6.2–10.6)	8.5 (6.2–10.7)	7.8 (6.3–9.9)	0.25
Signal intensity ratio LVOT/LV	0.92 (0.77–1.02)	0.93 (0.77–1.02)	0.90 (0.77–1.00)	0.34
Signal intensity ratio LVOT/LA	0.88 (0.72–1.00)	0.89 (0.72–1.00)	0.88 (0.69–0.97)	0.30
LV-aortic root angle (degrees)	126 (120–130)	126 (120–131)	126 (121–130)	0.89

Continuous variables are presented as median (25th–75th percentiles) and categorical variables as number (percentage). Group comparisons for continuous variables with Mann-Whitney U test and for categorical variables with chi-square test or Fisher’s exact test.

*AMVL* anterior mitral valve leaflet, *EDVi* end-diastolic volume indexed, *EF* ejection fraction, *ESVi* end-systolic volume indexed, *IVS* interventricular septum, *LA* left atrium, *LV* left ventricular, *LVOT* left ventricular outflow tract, *Minimum MV-IVS distance* minimum mitral valve leaflet tip to interventricular septum distance, *Minimum MV-IVSi distance* minimum mitral valve leaflet tip to interventricular septum distance indexed, *MV-IVS* mitral valve leaflet tip to interventricular septum distance, *MV-IVSi* mitral valve leaflet tip to interventricular septum distance indexed, *PMVL* posterior mitral valve leaflet, *SVi* stroke volume indexed

CMR was performed on a clinical 1.5T or 3T MRI system (n = 400 and n = 48, respectively; vendor: GE HealthCare, Milwaukee, USA, n = 321; Philips Healthcare, Best, Netherlands, n = 57; Siemens, Erlangen, Germany, n = 70). CMRs were scanned according to local protocols. Concerning the temporal resolution of the three-chamber bSSFP cine images, the median number of phases per cardiac cycle was 25 (25–75th percentile 24–30, range 20–45). The median reconstructed in-plane resolution was 1.2 mm (25–75th percentile 0.7–1.4 mm, range 0.6–2.0 mm) with a slice thickness of 8.0 mm (25–75th percentile 8.0–8.0, range 5.0–10.0 mm). CMR measurements are summarized in Table 1 and Supplementary Table S2.

**Fig. 3:**
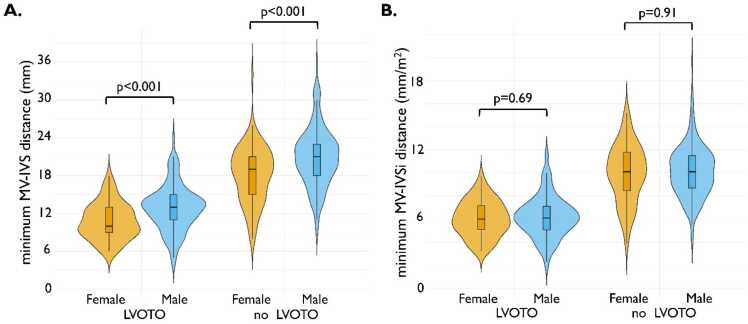
Comparison of the minimum distance between the mitral leaflet tip and the interventricular septum between hypertrophic cardiomyopathy patients with and without left ventricular outflow tract obstruction, stratified by sex. (A) Distance in mm, (B) distance indexed by body surface area in mm/m^2^. *LVOTO* left ventricular outflow tract obstruction, *Minimum MV-IVS distance* minimum mitral valve leaflet tip to interventricular septum distance, *Minimum MV-IVSi distance* minimum mitral valve leaflet tip to interventricular septum distance indexed

Of note, the MV-IVSi distance was smaller in the LVOTO group, irrespective of the cardiac phase in which it was measured. Indexing the MV-IVS distance to BSA accounted for both body size and sex differences. Specifically, in the LVOTO group, the median minimum MV-IVS distance was 13 (11–15) mm in males and 10 (9–13) mm in females (p < 0.001). However, after indexing to BSA, the median minimum MV-IVSi distance was 6.1 (5.1–7.1) mm/m^2^ in males and 6.0 (5.1–7.2) mm/m^2^ in females (p = 0.69) (Fig. 3).

Furthermore, the SI ratios of the LVOT/LV and LVOT/LA were lower in patients with LVOTO. Likewise, SAM of the mitral valve leaflets and flow artifacts in the LVOT during systole were more prevalent among patients with LVOTO.

### 4.2 Logistic regression and receiver operating characteristic curve analyses

**Fig. 4:**
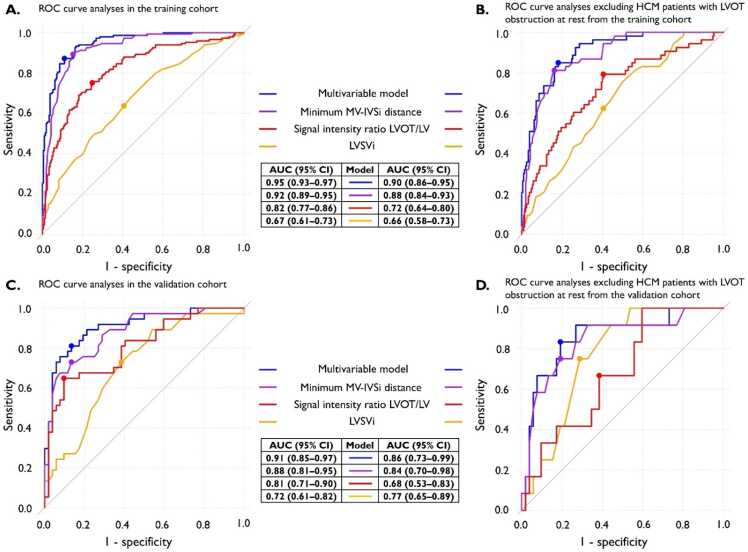
Receiver operating characteristic curve analyses of different cardiovascular magnetic resonance variables for discriminating left ventricular outflow tract obstruction on echocardiography. (A and C) Receiver operating characteristic curve analyses of the univariable and multivariable models developed and evaluated on the training cohort (A, n = 359) and validation cohort (C, n = 89); (B and D) Receiver operating characteristic curve analyses of univariable and multivariable models developed on the training cohort and evaluated on a subset of the training and validation cohort after excluding patients with LVOT obstruction at rest (B, training cohort, n = 263 and D, validation cohort, n = 64). *AUC* area under the curve, *CI* confidence interval, *LV* left ventricle, *LVOT* left ventricle outflow tract, *Minimum MV-IVSi distance* minimum mitral valve leaflet tip to interventricular septum distance indexed, *ROC* receiver operating characteristic, *SVi* stroke volume indexed

Table 2 presents the odds ratios and 95%CIs for all variables analyzed in univariable and multivariable models in the training cohort. The final model included as independent predictors: (1) the minimum MV-IVSi distance, (2) SI ratio LVOT/LV, and (3) LV stroke volume indexed to BSA. This multivariable model had excellent discriminatory ability with an AUC of 0.95 (95%CI, 0.93–0.97) in the training cohort and 0.91 (95%CI, 0.85–0.97) in the validation cohort (Fig. 4A and C). Notably, the univariable model of minimum MV-IVSi distance also showed very high diagnostic accuracy with AUCs of 0.92 (95%CI, 0.89–0.95) and 0.88 (95%CI, 0.81–0.95) in the training and validation cohorts, respectively.

**Table 2:** Univariable and multivariable logistic regression analyses in the training cohort of all the variables tested for discriminating left ventricular outflow tract obstruction at echocardiography (defined as ≥30 mmHg during rest or provocation).

	Univariable analysis		Multivariable analysis	
Variable	Odds ratio (95% CI)	p-value	Odds ratio (95% CI)	p-value
Interval between TTE and CMR (days)	1.00 (1.00–1.01)	0.45	–	
Age (y)	1.02 (1.00–1.03)	0.055	–	
Sex (male)	1.09 (0.69–1.73)	0.70	–	
Weight (kg)	1.01 (0.99–1.02)	0.24	–	
Height (cm)	1.00 (0.98–1.02)	0.83	–	
LVSVi (mL/m^2^)	1.07 (1.04–1.09)	<0.001	1.07 (1.04–1.12)	<0.001
Maximal wall thickness IVS (mm)	1.07 (1.02–1.12)	0.004	–	
Basal septal thickness (mm)	1.27 (1.17–1.39)	<0.001	–	
AMVL length (mm)	1.15 (1.08–1.22)	<0.001	–	
PMVL length (mm)	1.18 (1.09–1.28)	<0.001	–	
Aortic valve diameter (mm)	1.03 (0.97–1.11)	0.34	–	
Minimum MV-IVSi distance (mm/m^2^)	0.38 (0.31–0.45)	<0.001	0.42 (0.34–0.51)	<0.001
Presence of flow artifact	35.3 (19.5–67.9)	<0.001	–	
Systolic anterior motion of the mitral valve	20.5 (12.0–36.2)	<0.001	–	
Signal intensity ratio LVOT/LV	0.46 (0.39–0.55)*	<0.001	0.63 (0.50–0.78)*	<0.001
Signal intensity ratio LVOT/LA	0.43 (0.36–0.51)*	<0.001	–	
LV-aortic root angle (degrees)	0.99 (0.97–1.02)	0.70	–	

*CI* confidence interval, *CMR* cardiovascular magnetic resonance, *TTE* transthoracic echocardiography; others see Table 1

Data are presented as odds ratios with 95% confidence intervals.

* The odds ratio corresponds to a 0.1-unit change

To allow a direct comparison, an additional analysis was performed replacing the minimum MV-IVSi distance with the non-indexed minimum MV-IVS distance in the model-building process. Subsequently, the final multivariable model included as independent predictors: (1) the non-indexed minimum MV-IVS distance, (2) SI ratio LVOT/LV, (3) LV stroke volume indexed to BSA, and (4) sex. The corresponding odds ratios and 95%CIs are presented in Table S3. The non-indexed multivariable model had a similar performance to the indexed one with AUCs of 0.95 (95%CI, 0.92–0.97) in the training cohort and 0.93 (95%CI, 0.88–0.98) in the validation cohort. Similarly, the univariable model of non-indexed minimum MV-IVS distance showed performance closely matching that of the indexed distance, with AUCs of 0.91 (95%CI, 0.88–0.94) and 0.88 (95%CI, 0.82–0.95) in the training and validation cohorts, respectively.

### 4.3 Diagnostic thresholds of the minimum distance between the mitral leaflet tip and the interventricular septum

**Fig. 5:**
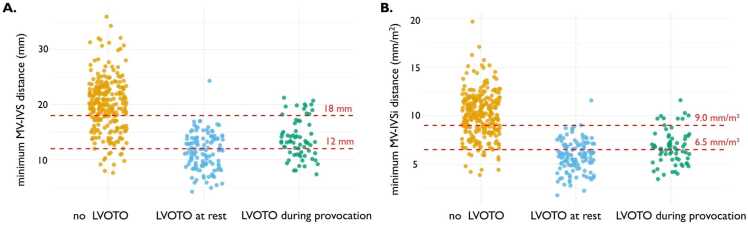
Distribution of the minimum distance between the mitral leaflet tip and the interventricular septum in the total population across three subgroups: no left ventricular outflow tract obstruction (LVOTO), LVOTO at rest, and LVOTO during provocation. (A) Distance in mm, (B) distance indexed by body surface area in mm/m^2^. *LVOTO* left ventricular outflow tract obstruction, *Minimum MV-IVS distance* minimum mitral valve leaflet tip to interventricular septum distance, *Minimum MV-IVSi distance* minimum mitral valve leaflet tip to interventricular septum distance indexed

Threshold analyses for both indexed and non-indexed minimum MV-IVS distances are presented in Table 3 and Supplementary Table S4. Cut-off values were selected to achieve a high positive predictive value (PPV) or high negative predictive value (NPV). For minimum MV-IVSi distance, a cutoff ≤6.5 mm/m^2^ yielded a sensitivity of 65%, specificity of 94%, and PPV of 89% for the detection of LVOTO in the validation cohort. Correspondingly, a distance of >9.0 mm/m^2^ had a sensitivity of 97%, a specificity of 56%, and an NPV of 97%. The distribution of the indexed and non-indexed minimum MV-IVS distance across patients with no LVOTO, LVOTO at rest, and LVOTO only during provocation are presented in Fig. 5. For the non-indexed minimum MV-IVS distance, thresholds of ≤12 and >18 mm yielded comparable discriminatory power (Table 3). The diagnostic performance in the training cohort is presented in the Supplementary Table S4.

**Table 3:** Diagnostic performance of the minimum indexed and non-indexed distance from the mitral valve leaflet tip to the interventricular septum for detecting left ventricular outflow tract obstruction at echocardiography (≥30 mmHg) in the validation cohort.

	Cut-off value	No LVOTO (n = 52)	LVOTO (n = 37)	Sensitivity (%)	Specificity (%)	PPV (%)	NPV (%)	Accuracy (%)
Minimum MV-IVS distance	≤12 mm	3 (6)	21 (57)	57	94	88	75	79
	>12 mm	49 (94)	16 (43)					
	≤18 mm	23 (44)	36 (97)	97	56	61	97	73
	>18 mm	29 (56)	1 (3)					
Minimum MV-IVS indexed distance	≤6.5 mm/m²	3 (6)	24 (65)	65	94	89	79	82
	>6.5 mm/m²	49 (94)	13 (35)					
	≤9.0 mm/m²	23 (44)	36 (97)	97	56	61	97	73
	>9.0 mm/m²	29 (56)	1 (3)					

Data are presented as number of patients (percentage) or percentage

*NPV* negative predictive value, *PPV* positive predictive value; others see Table 1

### 4.4 Subgroup analysis

The univariable and multivariable models developed on the complete training cohort were evaluated for detecting provocable only LVOTO (Fig. 4B and D). The AUCs were only slightly lower. For the multivariable model in the validation cohort, the AUC decreased to 0.86 (95%CI, 0.73–0.99), and in the univariable model of the minimum MV-IVSi distance, it decreased to 0.84 (95%CI, 0.70–0.98).

### 4.5 Intra- and interobserver variability

The results of the intra- and interobserver variability are presented in Table 4 and Supplementary Fig. S1. The minimum MV-IVSi distance had excellent ICCs of 0.98 for the intraobserver variability and 0.95 for the interobserver variability. The SI ratio LVOT/LV had an intra- and interobserver ICC of 0.89 and 0.74, respectively.

**Table 4:** Intra- and interobserver variability of the cardiovascular magnetic resonance variables.

	ICC or Cohen’s kappa	Bland-Altman analysis		
		Mean Difference	Upper LOA	Lower LOA
Intraobserver variability				
Maximal wall thickness IVS (mm)	0.98	−0.27	2.50	−3.04
Basal septal thickness (mm)	0.79	0.03	3.62	−3.56
AMVL length (mm)	0.73	1.80	8.28	−4.68
PMVL length (mm)	0.84	0.87	5.10	−3.37
Aortic valve diameter (mm)	0.93	0.07	1.64	3.28
Minimum MV-IVSi distance (mm/m²)	0.98	0.08	1.90	−1.74
Signal intensity ratio LVOT/LV	0.89	−0.02	0.18	−0.23
Signal intensity ratio LVOT/LA	0.94	−0.01	0.18	−0.21
LV-aortic root angle (degrees)	0.89	−1.87	7.09	−10.8
Presence of flow artifact	1.00	-	-	-
Systolic anterior motion of mitral valve	0.93	-	-	-
Interobserver variability				
Maximal wall thickness IVS (mm)	0.92	−1.80	2.76	−6.36
Basal septal thickness (mm)	0.62	−0.87	5.37	−7.10
AMVL length (mm)	0.63	0.50	7.65	−6.65
PMVL length (mm)	0.62	−0.10	6.75	−6.95
Aortic valve diameter (mm)	0.70	0.27	7.36	−6.83
Minimum MV-IVSi distance (mm/m²)	0.95	0.39	2.99	−2.22
Signal intensity ratio LVOT/LV	0.74	0.03	0.42	−0.37
Signal intensity ratio LVOT/LA	0.91	0.02	0.26	−0.22
LV-aortic root angle (degrees)	0.90	1.27	10.77	−8.24
Presence of flow artifact	0.93	-	-	-
Systolic anterior motion of mitral valve	0.93	-	-	-

*ICC* intraclass correlation coefficient, *LOA* limit of agreement; others see Table 1

Data are presented as intraclass correlation coefficients with corresponding Bland–Altman mean differences and limits of agreement for continuous variables and as Cohen’s kappa for binary variables.

## 5 Discussion

In this study, we demonstrate that (1) three CMR-derived parameters have an outstanding discriminatory ability with AUC of 0.91 in the validation cohort for detecting LVOTO, (2) the minimum MV-IVSi distance cut-off values of 6.5 and 9.0 mm/m^2^ provide a simple, reproducible metric for ruling in or ruling out LVOTO in HCM patients respectively, and (3) indexing MV-IVS distance to BSA accounts for both body size and sex differences, offering a more personalized assessment.

In sum, both the univariable model of the minimum MV-IVSi distance and the multivariable model perform very well in detecting LVOTO, with the first being much easier to use in clinical practice. Of note, only 4.3% of the CMRs were excluded due to poor imaging quality or incorrect three-chamber planning. This observation, in our view, underlies the feasibility of the proposed metric alongside its reliability, as underscored by the excellent intra- and interobserver agreement.

As mentioned before, morphological alterations of the LV and mitral valve contribute to LVOTO in HCM patients [9], [11], [12], [13], [19]. These alterations contribute to a reduction in the MV-IVS distance, facilitating SAM which results in LVOT narrowing, accelerating blood flow, and inducing a Venturi effect that further displaces the anterior mitral valve leaflet (AMVL) anteriorly, exacerbating obstruction [12], [19]. The MV-IVS distance has been previously assessed in relation to LVOT obstruction. Verheyen et al. demonstrated that end-systolic MV-IVS distance, measured by TTE, had an AUC of 0.91 (95%CI, 0.87–0.96) for discriminating LVOTO. Specifically, end-systolic MV-IVS distance ≤9 mm yielded a PPV of 92%, while >14 mm an NPV of 95% [9]. In another study of 1905 participants, machine learning methods were employed to identify 14 anatomical landmarks in the three-chamber cine images, resulting in 11 anatomical metrics [12]. Overall, mid-systolic MV-IVS had the strongest relationship with LVOT gradient and an AUC of 0.80 (95%CI, 0.77–0.82) [12]. We found AUCs of 0.92 (95%CI, 0.89–0.95) and 0.88 (95%CI, 0.81–0.95) in the training and validation cohorts, respectively. Importantly, in our analyses, we used the minimum distance of both mid- and end-systole and indexed for BSA.

Data presented in a recent study by Shiwani et al. regarding the diagnostic 15 mm threshold for HCM showed that not accounting for patient demographics can introduce biases [24]. Age, sex, and body size inﬂuence heart size, and a demographic-adjusted approach for LV hypertrophy improves diagnostic accuracy [24]. To extend this approach to our study, indexing to BSA normalizes values for differences in body size and, in our case, also accounts for sex differences. Although the diagnostic performance of the indexed and non-indexed minimum MV-IVS distances for predicting LVOTO was similar in our cohort, we favor the use of the indexed distance for clinical applications because sex differences were observed in our cohort as shown by our multivariate models.

We also measured the SI ratio LVOT/LV for assessing LVOTO in HCM. This measurement was used in line with a recent article by Vimalesvaran et al. in which they used the same method to assess aortic stenosis [23]. They found that the SI ratio of the aorta and LV shows a good correlation with aortic stenosis severity [23]. The turbulence and high velocity of the blood lead to spin dephasing, and consequently to signal loss on bSSFP images [23]. By analogy to this, patients with LVOTO exhibit a greater difference in SI between LVOT and LV, yielding consistently lower ratios. SI ratio LVOT/LV can be calculated easily and does not require additional imaging acquisition [23]. Ratios are used because absolute values can be affected by the different scanner vendors, acquisition protocols, and magnetic field strengths, introducing variability to the measurements [23].

SAM of the mitral valve leaflets, a well-established pathophysiologic mechanism of LVOTO, was a strong univariable predictor of LVOTO; however, it did not retain its predictive value in the final multivariable model. The role of LV-aortic root angle in the obstruction has also been reported in previous studies, in which steeper angles were associated with the presence of LVOTO [25], [26]. In contrast, in our cohort, no significant difference between patients with and without LVOTO was observed.

Other CMR studies have investigated alternative metrics for LVOTO. Two smaller studies (n = 92 and n = 46) reported that the LVOT/AO diameter ratio demonstrates promising diagnostic accuracy with an AUC of 0.90 and 0.87, respectively [17], [21]. Others proposed that the AMVL/LVOT diameter ratio may serve as a diagnostic metric [19], [20]. In one study, a ratio >2 was strongly associated with LVOTO [19], while in the other study, a ratio >2.3 yielded a sensitivity of 75% and a specificity of 85% [20]. Lastly, among 76 HCM patients of a case-control study, it was shown that the product IVSxAMVL had an AUC of 0.81 for detecting LVOTO [22]. In our study, we focused on the interplay between the tip of AMVL and IVS for narrowing LVOT rather than measuring the LVOT diameter. Moreover, AMVL length demonstrated low intra- and interobserver reproducibility, limiting its role as a diagnostic metric.

### 5.1 Clinical perspectives

CMR is currently recommended as part of the baseline assessment in HCM patients, providing a comprehensive evaluation of LV morphology and myocardial fibrosis. The proposed metrics can be easily integrated into standard CMR analysis, requiring only an additional minute for the measurement without the need for extra imaging acquisitions. The integration could reduce the need for further testing by TTE in a substantial number of HCM patients, leading to lower health care costs and greater patient convenience. Furthermore, evaluating the LVOT pressure gradient both at rest and under provocation is time-consuming and dependent on the expertise of the echocardiographer.

More specifically, the minimum MV-IVS distance could serve as a key predictor of LVOTO. A threshold of ≤6.5 mm/m^2^ strongly supports the presence of obstruction, while values >9.0 mm/m^2^ safely rule out LVOTO. For patients with values in the intermediate zone (6.5–9.0 mm/m^2^), further assessment with Doppler echocardiography is needed.

## 6 Limitations

Ideally, the study would have been prospective with patients undergoing CMR and Doppler echocardiography consecutively on the same day, as the LVOT gradient is influenced by loading conditions. Although detailed information on medication changes between echocardiography and CMR was not available for all patients, the proportion of patients with documented medication changes was negligible and did not significantly affect the overall conclusions. Moreover, the study was conducted in a single tertiary medical center, which may affect the generalizability of the outcome. Furthermore, no standard two-dimensional or four-dimensional flow measurements were performed using phase-contrast imaging with CMR. Therefore, we cannot compare our results to the direct velocity measurements which are also possible with CMR. Inter- and intraobserver variability for the measurements are presented and good, data on scan–rescan reproducibility are not available in our study. Moreover, all measurements were derived from a single standard three-chamber plane. A stack of three-chamber cine images was not available, precluding assessment of interslice reproducibility. Finally, no healthy controls were included, and thereby no normal reference values of the minimal MV-IVS distance could be established.

## 7 Conclusion

This study demonstrates that easy-to-measure and reproducible CMR-derived parameters can identify LVOTO in HCM patients. Particularly, the minimum MV-IVSi distance can accurately rule in or rule out LVOTO and easily be integrated into a standard CMR analysis.

## Funding

None.

## Author contributions

**Georgios M. Alexandridis:** Writing – review & editing, Writing – original draft, Visualization, Validation, Software, Resources, Project administration, Methodology, Investigation, Formal analysis, Data curation. **Stephan A.C. Schoonvelde:** Writing – review & editing, Writing – original draft, Supervision, Software, Resources, Methodology, Investigation, Data curation. **Anne J. Koppelaar:** Writing – review & editing, Writing – original draft, Validation, Software, Resources, Investigation, Data curation. **Peter-Paul Zwetsloot:** Writing – review & editing, Writing – original draft, Visualization, Supervision, Resources, Methodology, Investigation. **Alexander Hirsch:** Writing – review & editing, Writing – original draft, Visualization, Validation, Supervision, Software, Resources, Project administration, Methodology, Investigation, Formal analysis, Data curation, Conceptualization. **Michelle Michels:** Writing – review & editing, Writing – original draft, Visualization, Supervision, Resources, Methodology, Investigation, Conceptualization. **Ricardo P.J. Budde:** Writing – review & editing, Writing – original draft, Methodology, Investigation. **Isabella Kardys:** Writing – review & editing, Writing – original draft, Supervision, Software, Methodology, Formal analysis, Data curation. **Arend F.L. Schinkel:** Writing – review & editing, Writing – original draft, Methodology, Investigation. **Rudolf A. de Boer:** Writing – review & editing, Writing – original draft, Supervision, Resources, Methodology, Investigation.

## Declaration of competing interests

The Erasmus MC has received research grants and/or fees from Alnylam, AstraZeneca, Abbott, Bristol Myers Squibb, Novo Nordisk, Roche, and Siemens Healthineers; A. Hirsch received a research grant and consultancy fees from GE Healthcare and speaker fees from GE Healthcare, Bayer, Bristol Myers Squibb, GE Healthcare, Heartflow, and Siemens Healthineers. He is also a member of the medical advisory board of Medis Medical Imaging Systems and was MRI corelab supervisor of Cardialysis BV until 2022; R.A. de Boer has had speaker engagements with and/or received fees from and/or served on an advisory board for Abbott, AstraZeneca, Bristol Myers Squibb, Novo Nordisk, Roche, and Zoll; R.A. de Boer received travel support from Abbott and Novo Nordisk; P.-P. Zwetsloot is partially funded through a Dutch Heart Foundation Public Private Partnership Grant (CARMA, grant 01-003-2022-0358, Novo Nordisk, Dutch Heart Foundation) and has received speaker fees from MedNet, Pfizer, Bayer, Bristol Myers Squibb and consultancy fees from Bayer, Pfizer, PHARMO, Alnylam, Novo Nordisk, Cytokinetics and Bristol Myers Squibb; M. Michels receives research grants from Bristol Meyers Squibb and Cytokinetics and consultancy/speakers fees from Bristol Meyers Squibb, Cytokinetics, Sanofi, Alnylam and Bayer. All other authors declare no relevant disclosures in relation to this work.

## Data Availability

The data underlying this article will be shared on reasonable request to the corresponding author.
