# Supplementary material for: Cardiovascular magnetic resonance-derived metrics as diagnostic markers for left ventricular outflow tract obstruction in hypertrophic cardiomyopathy
Source: J Cardiovasc Magn Reson. 2026 Jan 14;28(1):102691. doi: 10.1016/j.jocmr.2026.102691 (PMC13168765; doi:10.1016/j.jocmr.2026.102691)
Supplement: Supplementary file 1 — Supplementary material [file mmc1.docx]

**Supplementary data**

**Figure S1.** Bland-Altman plots of the intra- and inter-observer agreement for the minimum indexed distance from the mitral leaflet tip to the interventricular septum and the signal intensity ratio of left ventricle outflow tract/left ventricle.


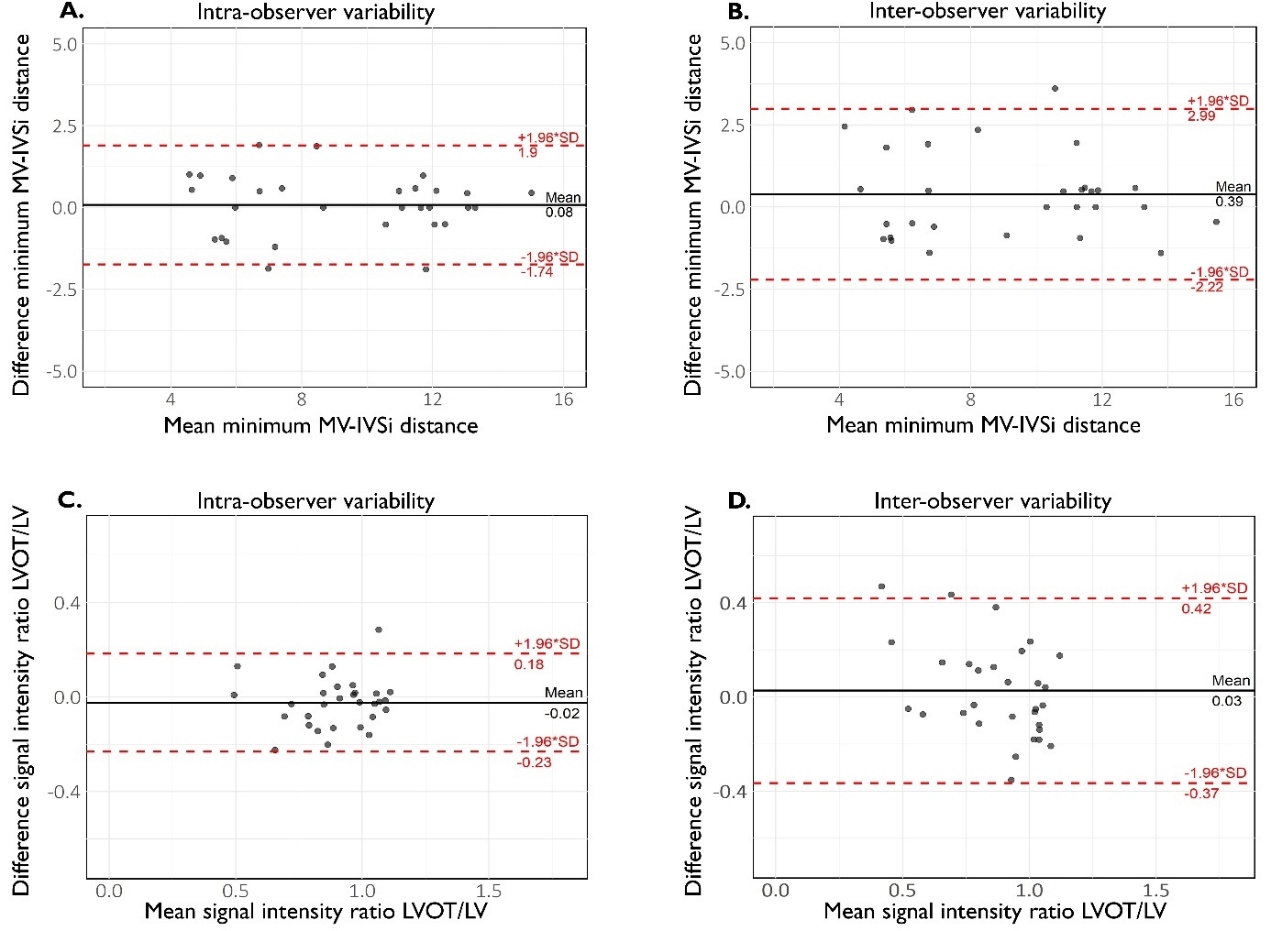


*Panel A.* Bland-Altman plot of the intra-observer agreement for minimum MV-IVSi distance; *Panel B.* Bland-Altman plot of the inter-observer agreement for minimum MV-IVSi distance; *Panel C.* Bland-Altman plot of the intra-observer agreement for signal intensity ratio LVOT/LV; *Panel D.* Bland-Altman plot of the inter-observer agreement for signal intensity ratio LVOT/LV; In each plot, the upper and lower red dashed lines correspond to the upper and lower limit of agreement respectively, while the black line corresponds to mean difference.

*Abbreviations*: LV=left ventricle; LVOT=left ventricle outflow tract; Minimum MV-IVSi distance=minimum mitral valve leaflet tip to interventricular septum distance indexed; SD=standard deviation

**Table S1.** Detailed definition of measured parameters in the 3-chamber view at cardiovascular magnetic resonance.

| Parameter | Definition |
| --- | --- |
| Aortic valve diameter | The aortic diameter measured in end-diastole at the level of the aortic valve, just below the sinuses of Valsalva |
| AMVL | The distance from the most distal extent of the leaflet to its insertion into the posterior aortic wall measured in diastole, while being fully extended parallel to the septum and the left ventricle free wall |
| Basal septal thickness | The thickness of the interventricular septum measured at the basal level of the heart in end-diastole |
| Flow artifacts | The presence of imaging distortions caused by the rapid movement of the blood resulting in decreased signal intensity/drop in signal during systole in LVOT |
| LV-aortic root angle | The angle between a line connecting the left ventricular apex to the midpoint of the mitral valve and a line passing through the center of the aortic root, measured in end-diastole |
| Maximal wall thickness IVS | The maximum thickness of the interventricular septum measured in end-diastole |
| MV-IVS | The distance from the mitral valve leaflet tip to interventricular septum measured in both mid-systole and end-systole |
| PMVL | The distance from the most distal extent of the leaflet to its insertion into the basal LV inferolateral free wall measured in diastole, while being fully extended parallel to the septum and the left ventricle free wall |
| SAM | The abnormal anterior displacement of the mitral valve leaflets toward the interventricular septum during systole |
| Signal intensity | The mean signal intensity was measured manually by selecting an area of approximately 0.5 cm^2^ in a region of interest at end-systole. The regions of interest that were measured are: left ventricular outflow tract, left ventricle, and left atrium 1-2 cm above the mitral valve. In cases of mitral regurgitation, the region of interest in the left atrium was adjusted to a different area, in order to avoid including the presence of backward blood flow due to mitral insufficiency |

*Abbreviations:* AMVL=anterior mitral valve leaflet; IVS=interventricular septum; MV-IVS=mitral valve leaflet tip to interventricular septum distance; PMVL=posterior mitral valve leaflet; SAM=systolic anterior motion of the mitral valve

**Table S2.** Diagnostic performance of the minimum indexed distance from the mitral valve leaflet tip to the interventricular septum and the presence of flow artifact for detecting left ventricular outflow tract obstruction at echocardiography defined as ≥30 mmHg.

|  | **Training cohort**  (n=359) | **No LVOT obstruction**  (n=210) | **LVOT  obstruction**  (n=149) | **P-value** | **Validation cohort**  (n=89) | **No LVOT obstruction**  (n=52) | **LVOT  obstruction**  (n=37) | **P-value** |
| --- | --- | --- | --- | --- | --- | --- | --- | --- |
| Age (years) | 54 (44–62) | 52 (43–62) | 55 (47–62) | 0.054 | 57 (48–63) | 57 (46–63) | 57 (50–63) | 0.59 |
| Male sex | 249 (69) | 144 (69) | 105 (71) | 0.79 | 52 (58) | 30 (58) | 22 (60) | 1.00 |
| Weight (kg) | 84 (74–94) | 83 (73–93) | 86 (75–95) | 0.20 | 83 (69–90) | 83 (70–93) | 83 (67–88) | 0.71 |
| Height (cm) | 176 (169–183) | 175 (169–183) | 178 (169–182) | 0.63 | 173 (165–181) | 173 (167–180) | 172 (160–183) | 0.46 |
| Body Surface Area (m^2^) | 2.0 (1.9–2.1) | 2.0 (1.9–2.1) | 2.0 (1.9–2.1) | 0.32 | 2.0 (1.8–2.1) | 2.0 (1.8–2.1) | 2.0 (1.7–2.1) | 0.68 |
| History of septal reduction therapy  (>3 months before imaging) | 16 (4) | 11 (5) | 5 (3) | 0.45 | 2 (2) | 2 (4) | 0 (0) | 0.51 |
| Time between CMR and echocardiography (days) | 60 (36–92) | 59 (38–90) | 63 (35–102) | 0.59 | 59 (28–92) | 66 (34–89) | 56 (24–101) | 0.42 |
| **Genotype testing** | (n=326) | (n=192) | (n=134) | 0.77 | (n=76) | (n=48) | (n=28) | 0.04 |
| (Likely) pathogenic gene variant | 175 (54) | 122 (64) | 53 (40) | <0.001 | 39 (57) | 27 (56) | 12 (43) | 0.37 |
| Gene |  |  |  | 0.99 |  |  |  | 0.09 |
| MYBPC3 | 124 (71) | 86 (70) | 38 (72) | – | 27 (69) | 17 (63) | 10 (83) | – |
| MYH7 | 23 (13) | 16 (13) | 7 (13) | – | 5 (13) | 5 (19) | 0 (0) | – |
| MYL2 | 9 (5) | 7 (4) | 2 (4) | – | 3 (8) | 1 (4) | 2 (17) | – |
| Other | 19 (11) | 13 (7) | 6 (11) | – | 4 (10) | 4 (15) | 0 (0) | – |
| **Transthoracic echocardiography** |  |  |  |  |  |  |  |  |
| LVOT gradient at rest (mmHg) | 8 (5–37) | 6 (4–8) | 49 (21–82) | <0.001 | 10 (6–45) | 6 (5–9) | 49 (23–64) | <0.001 |
| LVOT gradient at provocation (mmHg) | 13 (7–81) | 7 (5–10) | 88 (64–108) | <0.001 | 17 (7–67) | 8 (6–13) | 81 (61–100) | <0.001 |
| **Cardiovascular Magnetic Resonance** |  |  |  |  |  |  |  |  |
| LVEDVi (mL/m^2^) | 82 (72–94) | 80 (71–90) | 85 (74–98) | 0.003 | 80 (74–91) | 78 (73–86) | 82 (77–95) | 0.047 |
| LVESVi (mL/m^2^) | 32 (25–39) | 31 (26–39) | 32 (24–39) | 0.86 | 30 (26–36) | 31 (27–36) | 29 (25–38) | 0.61 |
| LVSVi (mL/m^2^) | 50 (44–57) | 48 (42–54) | 53 (47–60) | <0.001 | 50 (43–56) | 47 (41–53) | 53 (49–62) | 0.001 |
| LVEF (%) | 61 (56–66) | 60 (54–65) | 62 (57–68) | 0.001 | 62 (55–67) | 60 (55–65) | 65 (60–70) | 0.020 |
| LVEF <50% | 33 (9) | 26 (12) | 7 (5) | 0.022 | 7 (8) | 6 (12) | 1 (3) | 0.23 |
| Maximal wall thickness IVS (mm) | 18 (16–22) | 17 (15–21) | 19 (17–22) | <0.001 | 17 (15–20) | 17 (14–20) | 18 (16–20) | 0.32 |
| Basal septal thickness (mm) | 12 (10–14) | 11 (10–13) | 13 (11–15) | <0.001 | 11 (10–13) | 10 (9–12) | 11 (10–13) | 0.009 |
| Aortic valve diameter (mm) | 24 (21–26) | 24 (21–26) | 24 (22–26) | 0.39 | 24 (21–26) | 24 (21–25) | 24 (20–27) | 0.81 |
| Presence of flow artifact during systole in LVOT | 173 (48) | 40 (19) | 133 (89) | <0.001 | 46 (52) | 12 (23) | 34 (92) | <0.001 |
| Systolic anterior motion of the mitral valve | 139 (39) | 27 (13) | 112 (75) | <0.001 | 34 (38) | 7 (14) | 27 (73) | <0.001 |
| AMVL length (mm) | 27 (25–29.5) | 26 (24–28) | 28 (25–31) | <0.001 | 26 (23–29) | 26 (23–28) | 27 (25–30) | 0.039 |
| PMVL length (mm) | 15 (14–18) | 15 (13–17) | 16 (14–19) | <0.001 | 15 (13–18) | 15 (12–17) | 16 (14–19) | 0.032 |
| MV-IVS mid-systole (mm) | 18 (14–23) | 22 (19–25) | 13 (11–15) | <0.001 | 18 (13–22) | 21 (18–24) | 12 (10–15) | <0.001 |
| MV-IVSi mid-systole (mm/m^2^) | 9.3 (6.8–11.7) | 11.2 (9.7–12.9) | 6.6 (5.5–7.7) | <0.001 | 9 (6.6–11.2) | 10.8 (8.8–12.5) | 6.4 (5.0–7.0) | <0.001 |
| MV-IVS end-systole (mm) | 17 (13–22) | 21 (18–23) | 13 (11–15) | <0.001 | 16 (13–20) | 20 (15–22) | 14 (11–15) | <0.001 |
| MV-IVSi end-systole (mm/m^2^) | 8.8 (6.7–10.8) | 10.5 (9.0–11.7) | 6.6 (5.6–7.7) | <0.001 | 8.2 (6.8–10.1) | 9.7 (7.8–11.3) | 6.8 (5.7–8.0) | <0.001 |
| Minimum MV-IVS distance (mm) | 17 (13–21) | 20 (18–23) | 12 (10-14) | <0.001 | 15 (12–20) | 19 (15–22) | 12 (9–14) | <0.001 |
| Minimum MV-IVSi distance (mm/m^2^) | 8.5 (6.2–10.7) | 10.2 (8.8–11.6) | 6.1 (5.1–7.1) | <0.001 | 7.8 (6.3–9.9) | 9.5 (7.6–11.1) | 6.2 (5.0–7.1) | <0.001 |
| Signal intensity ratio LVOT/LV | 0.93 (0.77–1.02) | 0.99 (0.91–1.06) | 0.78 (0.64–0.90) | <0.001 | 0.90 (0.77–1.00) | 0.95 (0.86–1.06) | 0.75 (0.60–0.91) | <0.001 |
| Signal intensity ratio LVOT/LA | 0.89 (0.72–1.00) | 0.95 (0.88–1.06) | 0.71 (0.61–0.84) | <0.001 | 0.88 (0.69–0.97) | 0.93 (0.83–1.02) | 0.70 (0.59–0.84) | <0.001 |
| LV-aortic root angle (degrees) | 126 (120–131) | 126 (120–132) | 126 (120–130) | 0.76 | 126 (121–130) | 127 (121–130) | 124 (120–127) | 0.12 |

Continuous variables are presented as median (25^th^–75^th^ percentiles) and categorical variables as number (percentage).
Group comparisons for continuous variables with Mann-Whitney test and for categorical variables with Chi-square test or Fisher’s exact test.

*Abbreviations*: AMVL=anterior mitral valve leaflet; EDVi=end-diastolic volume indexed; EF=ejection fraction; ESVi=end-systolic volume indexed; IVS=interventricular septum; LA=left atrium; LV=left ventricular; LVOT=left ventricular outflow tract; Minimum MV-IVS distance=minimum mitral valve leaflet tip to interventricular septum distance; Minimum MV-IVSi distance=minimum mitral valve leaflet tip to interventricular septum distance indexed; MV-IVS=mitral valve leaflet tip to interventricular septum distance; MV-IVSi=mitral valve leaflet tip to interventricular septum distance indexed; PMVL=posterior mitral valve leaflet; SVi=stroke volume indexed

**Table S3.** Univariable and multivariable logistic regression analyses in the training cohort using the non-indexed minimum distance between the mitral leaflet tip and the intraventricular septum for discriminating left ventricular outflow tract obstruction at echocardiography (defined as ≥30 mmHg during rest or provocation).

|  | **Univariable analysis** | | **Multivariable analysis** | |
| --- | --- | --- | --- | --- |
| **Variable** | **Odds Ratio (95% CI)** | **p-value** | **Odds Ratio (95% CI)** | **p-value** |
| Sex (male) | 1.09 (0.69–1.73) | 0.70 | 3.80 (1.74–8.78 ) | 0.001 |
| LVSVi (ml/m^2^) | 1.07 (1.04–1.09) | <0.001 | 1.07 (1.03–1.11) | <0.001 |
| Minimum MV-IVS distance (mm) | 0.64 (0.58–0.69) | <0.001 | 0.66 (0.59–0.72) | <0.001 |
| Signal intensity ratio LVOT/LV | 0.46 (0.39–0.55)* | <0.001 | 0.63 (0.50–0.78)* | <0.001 |

*The Odds Ratio corresponds to a 0.1-unit change.

*Abbreviations*: CMR=cardiovascular magnetic resonance; TTE=transthoracic echocardiography; others see table 1

**Table S4.** Diagnostic performance of the minimum indexed and non-indexed distance from the mitral valve leaflet tip to the interventricular septum for detecting left ventricular outflow tract obstruction at echocardiography (≥30 mmHg) in the training and validation cohort.

|  |  | **Cutoff Value** | **no LVOTO (n=210)** | **LVOTO**  **(n=149)** | **Sensitivity** | **Specificity** | **PPV** | **NPV** | **Accuracy** |
| --- | --- | --- | --- | --- | --- | --- | --- | --- | --- |
| **Training cohort** | **Minimum**  **MV-IVS distance** | ≤12mm | 10 (5) | 75 (50) | 50% | 95% | 88% | 73% | 77% |
|  |  | >12mm | 200 (95) | 74 (50) |  |  |  |  |  |
|  |  | ≤18mm | 66 (31) | 140 (94) | 94% | 69% | 68% | 94% | 79% |
|  |  | >18mm | 144 (69) | 9 (6) |  |  |  |  |  |
|  | **Minimum**  **MV-IVS indexed distance** | ≤6.5mm/m² | 11 (5) | 92 (62) | 62% | 95% | 89% | 78% | 81% |
|  |  | >6.5mm/m² | 199 (95) | 57 (38) |  |  |  |  |  |
|  |  | ≤9.0mm/m² | 62 (30) | 141 (95) | 95% | 70% | 69% | 95% | 81% |
|  |  | >9.0mm/m² | 148 (70) | 8 (5) |  |  |  |  |  |
|  |  |  | **no LVOTO (n=52)** | **LVOTO**  **(n=37)** |  |  |  |  |  |
| **Validation cohort** | **Minimum**  **MV-IVS distance** | ≤12mm | 3 (6) | 21 (57) | 57% | 94% | 88% | 75% | 79% |
|  |  | >12mm | 49 (94) | 16 (43) |  |  |  |  |  |
|  |  | ≤18mm | 23 (44) | 36 (97) | 97% | 56% | 61% | 97% | 73% |
|  |  | >18mm | 29 (56) | 1 (3) |  |  |  |  |  |
|  | **Minimum**  **MV-IVS indexed distance** | ≤6.5mm/m² | 3 (6) | 24 (65) | 65% | 94% | 89% | 79% | 82% |
|  |  | >6.5mm/m² | 49 (94) | 13 (35) |  |  |  |  |  |
|  |  | ≤9.0mm/m² | 23 (44) | 36 (97) | 97% | 56% | 61% | 97% | 73% |
|  |  | >9.0mm/m² | 29 (56) | 1 (3) |  |  |  |  |  |

Data is presented as number of patients (percentage) or percentage.

*Abbreviations:* LVOTO=left ventricular outflow tract obstruction; Minimum MV-IVSi distance=minimum mitral valve leaflet tip to interventricular septum distance indexed; NPV=negative predictive value; PPV=positive predictive value
